# Masks-to-Skeleton: Multi-View Mask-Based Tree Skeleton Extraction with 3D Gaussian Splatting

**DOI:** 10.3390/s25144354

**Published:** 2025-07-11

**Authors:** Xinpeng Liu, Kanyu Xu, Risa Shinoda, Hiroaki Santo, Fumio Okura

**Affiliations:** Graduate School of Information Science and Technology, The University of Osaka, 1-5 Yamadaoka, Suita 565-0871, Osaka, Japan; liu.xinpeng@ist.osaka-u.ac.jp (X.L.); xu.kanyu@ist.osaka-u.ac.jp (K.X.); shinoda.risa@ist.osaka-u.ac.jp (R.S.); santo.hiroaki@ist.osaka-u.ac.jp (H.S.)

**Keywords:** tree skeletonization, point cloud, three-dimensional reconstruction, Gaussian splatting

## Abstract

Accurately reconstructing tree skeletons from multi-view images is challenging. While most existing works use skeletonization from 3D point clouds, thin branches with low-texture contrast often involve multi-view stereo (MVS) to produce noisy and fragmented point clouds, which break branch connectivity. Leveraging the recent development in accurate mask extraction from images, we introduce a mask-guided graph optimization framework that estimates a 3D skeleton directly from multi-view segmentation masks, bypassing the reliance on point cloud quality. In our method, a skeleton is modeled as a graph whose nodes store positions and radii while its adjacency matrix encodes branch connectivity. We use 3D Gaussian splatting (3DGS) to render silhouettes of the graph and directly optimize the nodes and the adjacency matrix to fit given multi-view silhouettes in a differentiable manner. Furthermore, we use a minimum spanning tree (MST) algorithm during the optimization loop to regularize the graph to a tree structure. Experiments on synthetic and real-world plants show consistent improvements in completeness and structural accuracy over existing point-cloud-based and heuristic baseline methods.

## 1. Introduction

Skeletal structures of plants (e.g., tree branches) encode the fundamental topology and geometry of plants [1] and play a crucial role in agricultural and botanical analysis. Accurately estimating plant skeletons benefits various downstream tasks, including high-throughput phenotyping [2,3,4,5,6], plant-organ segmentation [7,8], and agri-tech applications such as robotic pruning and fruit harvesting [9,10].

Traditional methods of plant skeletonization typically rely on high-fidelity 3D point cloud input captured by expensive sensing systems, such as light detection and ranging (LiDAR) and structured light scanners. While LiDAR offers millimeter-level precision, its high cost and operational complexity limit broader applicability [11,12]. In contrast, photogrammetry-based multi-view image methods offer a more accessible choice [13]. However, the branches’ characteristics of low-texture contrast and thin nature often cause multi-view stereo (MVS) to yield noisy and fragmented point clouds, in which branches are often missing or disconnected.

Existing methods of tree skeleton estimation from 3D point clouds typically follow a two-stage routine: first, extracting an initial skeleton from the point clouds and then refining connectivity using heuristic or learning-based rules. Current approaches fall into three families. Among them, particle flow-based models require heavy parameter tuning [14,15,16]. Geometry-based methods, including thinning [17,18,19], clustering [20,21,22], and minimum spanning tree (MST) refinement [23,24,25], are effective under clean and complete data. Recent deep-learning methods like Smart-Tree and TreePartNet [22,26] demand large labelled sets and struggle with real-world noise.

To overcome the limitations of point-cloud-based skeletonization, we propose a mask-guided graph optimization framework that estimates a 3D tree graph structure directly from multi-view segmentation masks. We represent the skeleton as a graph, where nodes have their position and radii of branches, and the edges are represented as an adjacency matrix.

Given the rough initial graph extracted from MVS-based point clouds, we optimize it via 3D Gaussian splatting (3DGS) [27], projecting the graph skeleton onto the image plane. We minimize a multi-view silhouette loss so that the graph fits the observed silhouettes. The inherent blur of 3D Gaussians achieves robustness to appearance differences among views [27], resulting in camera pose errors and mask errors in our case.

Our method stands on the recent notable development of segmentation algorithms, where yielding accurate 2D masks is essential for guiding the mask-based refinement of the 3D skeleton. Currently, no segmentation model is trained explicitly for tree branches, especially under natural scenes’ diverse lighting and occlusion conditions. We, therefore, combine a recent language-grounded segmentation model, Lang-SAM (Language segment-anything, https://github.com/luca-medeiros/lang-segment-anything (accessed on 10 May 2025)), and an accurate segmentation model, BiRefNet-HQ [28], for acquiring accurate segmentation of tree branches.

**Figure 1:**
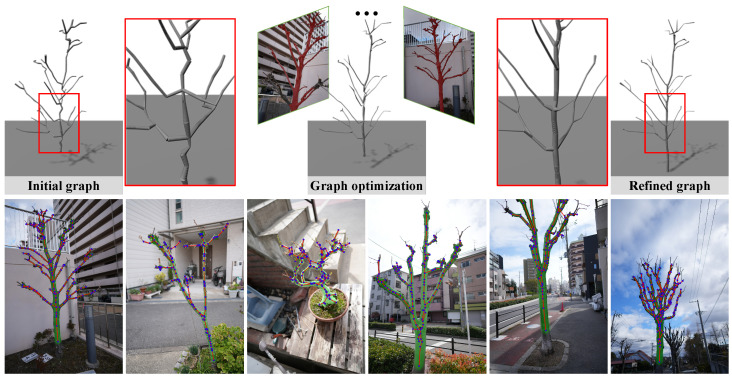
We reconstruct 3D branch structures using refined masks from multiple views, as shown in red. Transparent green overlays indicate the predicted tree shape and branch radii. Blue points and red lines represent graph nodes and branch connections, respectively.

Figure 1 illustrates the visual results of our method on real trees, where green regions represent predicted branches with their radii, blue points denote graph nodes, and red lines indicate branch connections, highlighting that our method faithfully extracts the graph structure as well as the tree shape as the nodes’ radii.

Our key contribution is introducing a novel mask-guided optimization framework that estimates 3D tree-structured skeletons. By leveraging differentiable 3DGS, our method transforms the graph into soft silhouettes and aligns them with the multi-view masks. This design enables the model to mitigate pose and segmentation noise, yielding topologically valid and geometrically accurate skeletons without requiring point-cloud-specific tuning, as shown in our experiments comparing our method with the state-of-the-art tree skeletonization methods. Our implementation is available in the public GitHub repository (https://github.com/huntorochi/Masks-to-Skeleton, accessed on 18 May 2025).

## 2. Related Work

### 2.1. Three-Dimensional Tree Skeleton Extraction

Estimating the skeletal structure of plants and trees is a long-lived task across computer vision (CV) and plant sciences [29]. For tree skeleton extraction, most approaches rely on 3D point clouds observed by LiDAR [20,30] or MVS [4,13]. Regardless of the capturing methods, most methods follow a two-stage pipeline [31]: (1) initial skeletonization to obtain preliminary branch structures and (2) graph refinements, such as using MST or shortest-path algorithms (e.g., Dijkstra’s algorithm), to optimize connectivity and topology [1,8,24,32,33].

#### 2.1.1. Particle-Flow Modeling

A traditional approach to generating plausible skeletons is to simulate particle trajectories guided by physical rules or probabilistic distributions [14,15,16]. Although effective in reconstructing missing branches, these methods are susceptible to parameter tuning and require trees to exhibit strong structural self-similarity [14,16].

#### 2.1.2. Geometry-Based Methods

Most methods directly use the geometry of point clouds to refine the tree structure, such as voxel thinning [17,18], clustering-based skeleton extraction [20,21,22,34], and spanning tree methods [23,24,25]. Thinning methods iteratively erode the data volume but are prone to errors under noisy or incomplete conditions [17,19,35]. Clustering methods segment point clouds into smaller components before linking them into a coherent skeleton, requiring precise neighborhood radius selection, but typically struggle with occluded or sparsely sampled data [20,21]. Spanning tree-based methods [23,24,25] guarantee connectivity through global optimization but cannot recover missing branches absent from the initial point cloud. Their pruning thresholds are often species-specific.

#### 2.1.3. Learning-Based Methods

Recent advancements in deep learning have introduced neural approaches for skeletonization. For example, Smart-Tree [26] employs sparse voxel convolutional neural networks to predict medial axes, improving accuracy around complex branch structures. Similarly, TreePartNet [22] decomposes point clouds into generalized cylinders using semantic segmentation and neural clustering modules. Although these methods achieve stronger generalization across tree species, they rely on large-scale supervised training. Moreover, their performance degrades due to a domain gap between synthetic training and real-world inference [36,37].

To address the limitations of point-cloud-based approaches, we introduce the first attempt at a mask-guided graph optimization framework for tree skeleton extraction, which refines connectivity through differentiable rendering to fit the multi-view segmentation masks.

### 2.2. Image-Guided 3D Structure Reconstruction via Differentiable Rendering

Although in different contexts, recent sketch-based 3D reconstruction methods yield 3D line-like structures from 2D supervision using compact primitives such as Bézier curves [38], superquadric strokes [39], or learned sketch embeddings [40]. Among them, Diff3DS [38] is the most relevant to our task, which represents objects as 3D Bézier curves and projects them onto multi-view images using a differentiable renderer based on DiffVG [41]. The silhouettes are denoised using a pre-trained diffusion model. While these methods are helpful for sketch-based reconstruction, these primitives lack explicit connectivity and topological structure, making it challenging to reconstruct complete tree skeletons as a graph structure.

Compared with sketch-based methods, our key is to use a graph-based representation, where branch connectivity and junctions are encoded explicitly. We directly optimize the whole graph structure via differentiable rendering of 3D Gaussian primitives, supporting soft silhouette rendering with spatially varying blur.

## 3. Method

**Figure 2:**
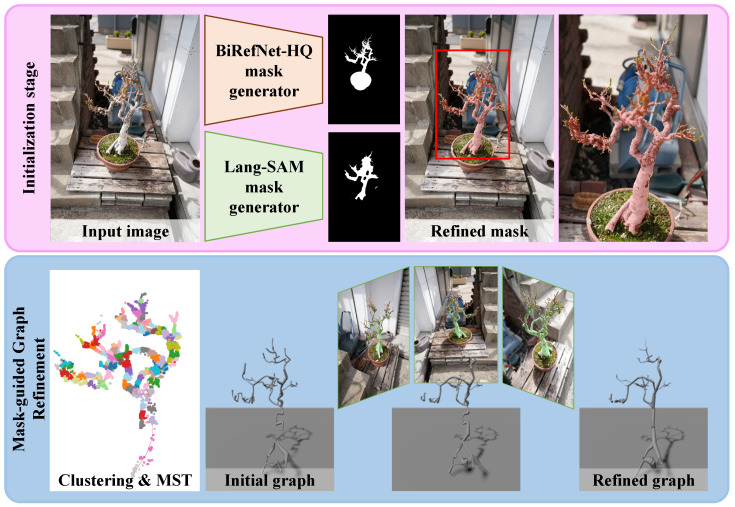
Pipeline overview. The provided input images, two complementary mask generators, BiRefNet-HQ for high-resolution foreground masks, and Lang-SAM for language-guided object masks are fused into refined masks. We use the MST and clustering to obtain an initial graph. Finally, the initial graph is refined through multi-view masks, producing a 3D plant skeleton that is guaranteed to be a valid tree.

Our framework estimates accurate 3D plant skeletons from multi-view images, as shown in Figure 2. We first extract segmentation masks from multi-view images using the Lang-SAM and BiRefNet-HQ [28], which complementarily extract the semantic region information and fine details. We then initialize a rough tree graph structure by clustering the MVS-based 3DGS point cloud and the MST constraint. We convert the graph structure into Gaussian primitives for differentiable graph rendering and optimize the graph components with silhouette losses while considering tree structure validity.

### 3.1. Preprocessing and Initialization

This section comprises two main components: (1) multi-model mask extraction and (2) graph initialization from filtered point clouds.

#### 3.1.1. Mask Extraction

While our goal is not to propose a new segmentation method, obtaining a high-quality segmentation mask of trees is essential for supervising the graph optimization process described later. Accurate segmentation of plant masks in natural scenes has been challenging due to uncontrolled illumination and background clutter; however, we found that the recent development of image segmentation methods using foundation models enables reasonable segmentation of natural trees.

We adopt a two-stage strategy that combines the strengths of two foundation models: BiRefNet-HQ, a high-resolution foreground segmentation model, and Lang-SAM, a language-grounded model guided by text prompts. The fusion addresses the limitations of each model. BiRefNet-HQ generates spatially accurate masks but struggles to distinguish tree-only foreground from background clutter, while Lang-SAM lacks sufficient spatial resolution to capture fine structural details.

**Figure 3:**
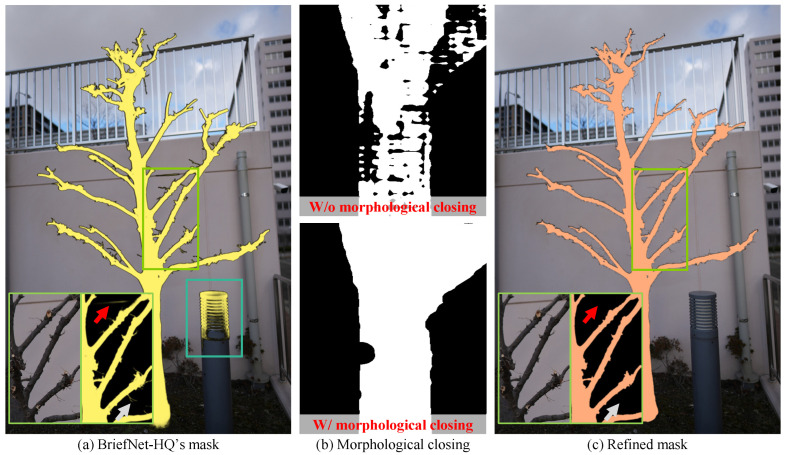
Mask fusion pipeline. (**a**) The output of BirefNet-HQ retains fine structures but includes background noise (see light blue box). (**b**) Lang-SAM prediction before (top) and after (bottom) morphological closing, where closing fills small gaps. (**c**) The refined mask is obtained by combining both predictions, applying morphological filtering and connected component analysis to preserve only the main plant region. As a result, small background artifacts (red arrows) and ultra-thin branches (gray arrows) are effectively suppressed.

Specifically, given an RGB input image $I\;\in \;R^{H\times W\times 3}$, we first generate a foreground alpha mask $M_{B}\;\in \;{\left\{0,1\right\}}^{H\times W}$ with BiRefNet-HQ, which may contain objects other than the target tree but are judged as the foreground, as shown in Figure 3a. To suppress the leakage, we apply Lang-SAM with the text prompt “tree” to obtain the rough area of the target tree $M_{L}\;\in \;{\left\{0,1\right\}}^{H\times W}$. Then, this is followed by the morphological closing Close(·,s) with a kernel size of s=7 to reduce fragmentation artifacts, as shown in Figure 3b. Formally,

(1) $${\tilde{M}}_{L}=\mathrm{Close}\left(M_{L},\;s\right).$$

The final mask is computed by intersecting both masks and retaining only the largest connected component (see Figure 3c) as follows:

(2) $$M_{\mathrm{final}}=\mathrm{LargestCC}\;\left(M_{B}\;\wedge \;{\tilde{M}}_{L}\right),$$

where ∧ denotes element-wise logical AND, and LargestCC only retains the largest connected component.

#### 3.1.2. Graph Initialization

In urban or roadside scenes, the camera motion is often restricted to part of the whole hemisphere, resulting in noisy and fragmented point clouds by MVS. We thus use 3DGS initialized by the MVS point cloud to further densify the points. Since both clouds often include unreliable outliers, we apply a multi-view mask consistency filtering, i.e., projecting each point into all views and retaining it only if its projection lies at least $N_{\min}$ foreground masks.

Let $P_{\mathrm{MVS}}=\left\{p_{i}\in R^{3}\right\}$ and $P_{\mathrm{GS}}=\left\{p_{i}\in R^{3}\right\}$ denote the filtered point clouds from MVS and 3DGS, respectively. We then yield the dense point cloud as follows:

(3) $$P_{\mathrm{init}}=P_{\mathrm{MVS}}\cup P_{\mathrm{GS}}.$$

We clean $P_{\mathrm{init}}$ using radius-based and statistical outlier removal, then apply *K*-means clustering. Let a tuple $v_{i}=\left(c_{i},r_{i}\right),\left(1\leq i\leq K\right)$ contain a cluster centroid $c_{i}\in R^{3}$ and cluster’s median local radius $r_{i}\in R_{+}$. We connect the nodes using an MST algorithm, where the edge cost is defined as the Euclidean distance between $c_{i}$, yielding the initial graph:

(4) $$G_{\mathrm{init}}=\left(V,\;E_{\mathrm{init}}\right)=\left({\left\{\;\left(c_{i},r_{i}\right)\;\right\}}_{i=1}^{K},\;E_{\mathrm{init}}\right),$$

where each node $v_{i}$ encodes the position and radius, and each edge $\left(i,j\right)\in E_{\mathrm{init}}$ reflects a branch connection determined by the MST.

To enable the gradient-based optimization of the adjacency structure, we represent the graph connectivity using a differentiable adjacency matrix $A\in R^{K\times K}$. Specifically, we initialize adjacency probabilities with values close to 0 or 1 based on initial edges as follows:

(5) $$A_{ij}=\left\{\begin{array}{cc} 1-\epsilon , & \left(i,j\right)\in E_{\mathrm{init}}\\ \epsilon , & \left(i,j\right)\notin E_{\mathrm{init}} \end{array}\right.,$$

where $\epsilon =10^{-6}$ prevents gradient saturation. To facilitate optimization, we reparameterize the adjacency probabilities using the inverse sigmoid transformation:

(6) $$A_{\mathrm{init}}=\mathrm{logit}\left(A\right)=\log\left(\frac{A}{1-A}\right).$$

### 3.2. Mask-Guided Graph Refinement

#### 3.2.1. Three-Dimensional Gaussian Splatting

Three-dimensional Gaussian splatting, introduced by Kerbl et al. [27], represents a scene as a set of anisotropic 3D Gaussians, facilitating efficient differentiable rendering. Each Gaussian primitive *G* is parameterized by a 3D center $\mu \in R^{3}$ and a covariance matrix $\Sigma \in R^{3\times 3}$:

(7) $$G\left(p\right)=e^{-\frac{1}{2}{\left(p-\mu \right)}^{⊤}\Sigma^{-1}\left(p-\mu \right)},$$

where $p\in R^{3}$ denotes any 3D spatial location. The covariance matrix Σ is decomposed into a scaling matrix $S=\mathrm{diag}(s_{1},s_{2},s_{3})$ and a rotation matrix R∈SO(3) as follows:

(8) $$\Sigma =RSS^{⊤}R^{⊤}.$$

In the 3DGS implementation, scale and rotation are represented by a scaling vector $s={\left[s_{1},s_{2},s_{3}\right]}^{⊤}\in R_{\geq 0}^{3}$ and the quaternion form q, respectively. Both parameters are optimized independently in the pipeline along with the optimization of Gaussian appearances {o,c} containing opacity *o*, and color c is represented as its RGB direct component (DC) and view-dependent spherical harmonics coefficients. The projected opacity α of a Gaussian at a pixel location is as follows:

(9) $$\alpha =1-e^{-o\cdot G(p)}.$$

In our pipeline, we specifically exploit opacity values to encode the probability of structural edges, which is detailed in the following section.

#### 3.2.2. Mask-Guided Graph Refinement

Our key contribution is formulating the structural refinement of plant skeleton graphs as a self-supervised optimization problem using multi-view silhouette masks. Starting from the initial graph, we render Gaussian samples along each edge defined by the graph edges.

Formally, given an edge connecting node $v_{i}$ and $v_{j}$ with respective radii $r_{i}$ and $r_{j}$, we uniformly sample points along the edge to create Gaussian primitives:

(10) $$p_{n}^{\left(ij\right)}=c_{i}+\frac{n}{N}\left(c_{j}-c_{i}\right),\;r_{n}^{\left(ij\right)}=r_{i}+\frac{n}{N}\left(r_{j}-r_{i}\right),\;n=0,\ldots ,N,$$

where *N* denotes the number of sampled Gaussians per edge, ensuring dense coverage (we use *N* = 64 in all experiments).

To align the graph structure with silhouette masks, we initialize each Gaussian with isotropic scale parameters $\left(s_{x},s_{y},s_{z}\right)=\left(r_{n}^{\left(ij\right)},r_{n}^{\left(ij\right)},r_{n}^{\left(ij\right)}\right)$ and set the rotation and color parameters to fixed constants, which are not updated during optimization.

We explicitly parameterize edge existence probabilities using the differentiable adjacency matrix A defined earlier. The opacity of Gaussian primitives sampled along an edge (i,j) is thus directly tied to the adjacency probability $A_{ij}$ as follows:

(11) $$o_{k}^{\left(ij\right)}=A_{ij}=\gamma \left(A_{init}^{\left(ij\right)}\right),$$

where γ(·) denotes the sigmoid activation.

During rendering, pixel-level opacity α is computed using the accumulation of projected Gaussian opacities, yielding a differentiable mask:

(12) $$\alpha \left(x\right)=1-\prod_{g\in G\mathrm{proj}(x)}\left(1-o_{g}\cdot G_{g}\left(x\right)\right),$$

where $G_{\mathrm{proj}}\left(x\right)$ is the set of Gaussians projected onto pixel x.

### 3.3. Structure-Aware Graph Optimization

Our framework refines both the topology and geometry of the plant skeleton graph through two complementary objectives:

(13) $$L_{\mathrm{total}}=L_{\mathrm{silhouette}}+L_{\mathrm{graph}}.$$

#### 3.3.1. Silhouette Supervision

As described above, we render the graph structure using 3DGS and obtain a soft silhouette mask α(x). We supervise this mask using the binary foreground segmentation mask M(x) extracted from multi-view images.

To guide the adjacency matrix A toward tree-like connectivity, we introduce a tree graph prior via a differentiable selective feature suppression (SFS) layer proposed in TreeFormer [42]. This layer converts an arbitrary graph to an MST-based tree graph in a differentiable manner, and thus, we convert the adjacency matrix A to the one representing an MST, $A_{\mathrm{mst}}$. We then obtain the MST graph’s soft silhouette mask $\alpha_{\mathrm{mst}}\left(x\right)$ in the same manner as computing the soft silhouette mask α(x).

The final silhouette loss to minimize is as follows:

(14) $$L_{\mathrm{silhouette}}=\lambda_{\mathrm{GS}}\cdot L_{\mathrm{GS}}+\lambda_{\mathrm{MST}}\cdot L_{\mathrm{MST}},$$

where

(15) $$L_{\mathrm{GS}}=\frac{1}{\left|\Omega \right|}\sum_{x\in \Omega }\left|\alpha \left(x\right)-M\left(x\right)\right|,$$

(16) $$L_{\mathrm{MST}}=\frac{1}{\left|\Omega \right|}\sum_{x\in \Omega }\left|\alpha_{\mathrm{mst}}\left(x\right)-M\left(x\right)\right|,$$

and Ω denotes the set of foreground-relevant pixels across all views, i.e., pixels within the union of all refined masks.

This setup encourages A to remain fully differentiable while being softly aligned with a plausible tree topology through silhouette supervision.

#### 3.3.2. Graph Geometry Regularization

To encourage physically plausible and topologically meaningful structures, we incorporate five regularization terms inspired by trees’ characteristics:

##### Repulsion Loss $L_{\mathrm{rep}}$

Repulsion loss penalizes overly close node pairs using a Gaussian kernel:

$$L_{\mathrm{rep}}=\sum_{i<j}e^{-\frac{∥c_{i}-c_{j}{∥}^{2}}{\sigma_{\mathrm{rep}}^{2}}}.$$

##### Edge Length Loss $L_{\text{edge-short}}$

Edge length loss penalizes short edges to suppress trivial links:

$$L_{\text{edge-short}}=\sum_{(i,j)\in E}e^{-\frac{∥c_{i}-c_{j}{∥}^{2}}{\sigma_{\mathrm{edge}}^{2}}}.$$

##### Angle Fold Loss $L_{\mathrm{angle}}$

Angle fold loss penalizes sharp back-and-forth turns (i.e., zig-zag edges) by enforcing a minimum angle between consecutive edges. For each triple (i,j,k) sharing a common pivot node *j*, we compute the angle $\theta_{ijk}$ formed between edges $E^{\left(ij\right)}$ and $E^{\left(jk\right)}$, and apply the following:

$$L_{\mathrm{angle}}=\sum_{\left(i,j,k\right)}\mathrm{softplus}\left(\cos\theta_{ijk}-\cos\theta_{\min}\right).$$

##### Midpoint Direction Loss $L_{\mathrm{mid}}$

Midpoint direction loss penalizes directionally aligned edges that are spatially too close. For each edge pair (i,j,k) that shares a common node *j*, we consider the pair of edges $E^{\left(ij\right)}$ and $E^{\left(jk\right)}$, and apply the loss if they are nearly collinear:

$$L_{\mathrm{mid}}=\sum_{\left(i,j,k\right)}\mathrm{softplus}\left(∥m^{\left(ij\right)}-m^{\left(jk\right)}{∥}_{2}-d_{\max}\right),$$

where $m^{\left(ij\right)}=\frac{1}{2}\left(c_{i}+c_{j}\right)$ and $m^{\left(jk\right)}=\frac{1}{2}\left(c_{j}+c_{k}\right)$ denote the midpoints of the two edges. We consider the pair only if the angle between the two edges satisfies the following:

$$\cos\left(\theta_{ijk}\right)=\frac{{\left(c_{j}-c_{i}\right)}^{⊤}\left(c_{k}-c_{j}\right)}{∥c_{j}-c_{i}∥\cdot ∥c_{k}-c_{j}∥}>\tau_{\mathrm{dir}}.$$

##### Radius Lower Bound Loss $L_{\mathrm{radius}}$

Radius lower bound loss prevents branch radii from collapsing:

$$L_{\mathrm{radius}}=\sum_{i\in E}\mathrm{softplus}\left(r_{\min}-r_{i}\right).$$

We combine these terms into the full geometry-aware loss:

(17) $$\begin{array}{cc} L_{\mathrm{graph}}= & \lambda_{\mathrm{rep}}L_{\mathrm{rep}}+\lambda_{\text{edge-short}}L_{\text{edge-short}}+\lambda_{\mathrm{angle}}L_{\mathrm{angle}}\\ \; & +\;\lambda_{\mathrm{mid}}L_{\mathrm{mid}}+\lambda_{\mathrm{radius}}L_{\mathrm{radius}}. \end{array}$$

Together, these modules form a fully differentiable pipeline that transforms raw 2D masks into a geometry- and topology-consistent 3D tree structure without the need for dataset-specific tuning or post-processing heuristics. The hyper-parameter settings are summarized in Section 4.1.

### 3.4. Overall Algorithm

To clearly illustrate our mask-guided tree skeleton reconstruction pipeline, we summarize our overall method in Algorithm 1.
**Algorithm 1:** Mask-Guided Tree Skeleton Reconstruction.
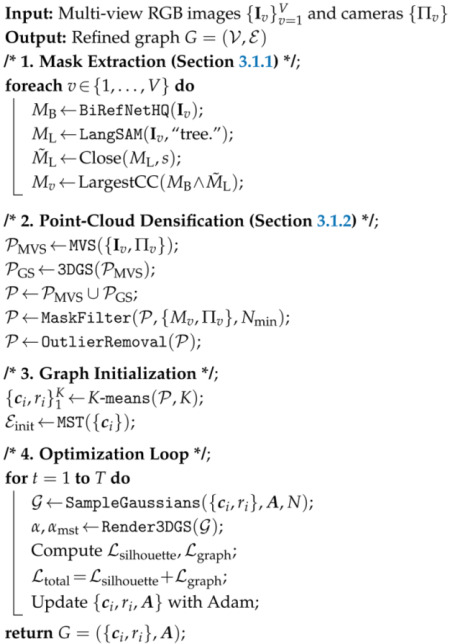


## 4. Experiments

### 4.1. Implementation Details

We implement our method with PyTorch (v2.6.0) and 3DGS’s CUDA kernels for rasterization. All experiments were conducted on a Windows workstation equipped with an AMD 3970X CPU and an NVIDIA RTX 4090 GPU. Our method is implemented as a modular PyTorch framework, utilizing the gsplat rasterizer [43] for rendering.

#### Hyper-Parameters

We summarize all hyper-parameters used in the silhouette supervision and geometry-aware optimization. Unless otherwise specified, the following values are fixed throughout all experiments.

For silhouette loss, we set $\lambda_{\mathrm{GS}}=0.1$ and $\lambda_{\mathrm{MST}}=1.0$. For geometry regularization, we fix all weights $\lambda_{*}=0.05$ and use thresholds $\sigma_{\mathrm{rep}}=2\;\mathrm{cm}$, $\sigma_{\mathrm{edge}}=2\;\mathrm{cm}$, $\theta_{\min}=20^{\circ }$, $\tau_{\mathrm{dir}}=0.90$, $d_{\max}=2\;\mathrm{cm}$, and $r_{\min}=1\;\mathrm{cm}$.

### 4.2. Dataset

**Figure 4:**
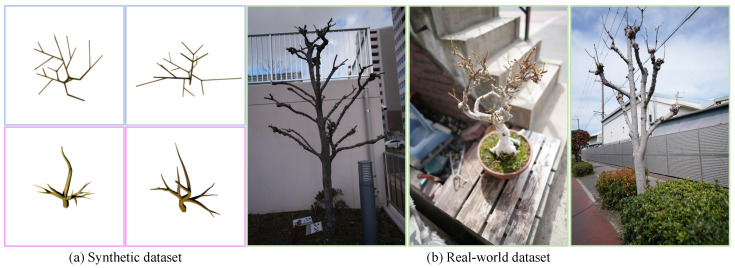
Datasets used in our experiments. (**a**) Synthetic dataset. Blue boxes highlight L-system-based trees characterized by relatively straight and uniform branches, while pink boxes highlight Mtree-based trees exhibiting more pronounced curvature and significant diameter variation. (**b**) Real-world dataset. Images of urban and roadside trees were captured from the sidewalk side to minimize background clutter and occlusion.

We evaluate our method using two datasets that reflect both controlled and real-world conditions: one synthetic (hereafter referred to as the *synthetic dataset*) and one real-world (the *real-world dataset*), as illustrated in Figure 4.

We construct two subsets for synthetic data to reflect different modeling challenges. We build the first subset using a 3D extension of the L-system-based tree generation method from TreeFormer [42]. While the original method generates 2D tree structures for image annotation, we adapt it to produce procedurally generated 3D trees. This enables full control over structural parameters and access to ground-truth topology for quantitative evaluation. We generate 50 random trees with varying branching patterns and morphological complexity. These L-system trees are designed at a moderate scale, approximately 2–5 m in height, matching typical real-world sampling sizes.

To further diversify the dataset and introduce more complex structures, we additionally generate a second synthetic subset using a modular tree framework, Mtree (https://github.com/MaximeHerpin/modular_tree (accessed on 10 May 2025)). Similarly, we generate 50 random Mtree trees reaching up to 20 m in height. These trees exhibit significantly more curved and organically shaped branches, with pronounced diameter variations from trunk to terminal branches. These characteristics present a more challenging testbed for our method, particularly in accurately predicting branch radii.

Our real-world dataset consists of urban and roadside vegetation captured using a Sony α7R V camera (Sony Group Corporation, Tokyo, Japan) at a nominal resolution of 9352×6254 pixels. The scenes include approximately 6 trees, comprising common street-side landscaping trees and ornamental species. Image capture is performed from the sidewalk side to avoid occlusions and suppress background clutter, resulting in partial views and permanent self-occlusions. These conditions introduce substantial visual challenges, including occluded branches, uneven lighting, and non-uniform background textures, thereby simulating practical constraints in real-world reconstruction tasks. We also note that some tree species exhibit anatomical irregularities, such as galls or nodal swellings, that introduce additional complexity during reconstruction.

### 4.3. Evaluation Metrics

We use complementary metrics to evaluate both spatial accuracy and topological fidelity of the predicted plant skeletons.

#### 4.3.1. Chamfer Distance

To assess geometric similarity, we compute the bidirectional Chamfer distance between the predicted and ground-truth skeletons. For each skeleton, we sample points uniformly along every edge using fixed-length segments. Specifically, we use a spacing of 1mm for L-system trees and 1cm for Mtree trees, reflecting the scale differences between the two synthetic subsets. Let $P_{\mathrm{pred}}$ and $P_{\mathrm{gt}}$ denote the two resulting point clouds. The Chamfer distance is computed as follows:

(18) $$\mathrm{CD}\left(P_{\mathrm{pred}},P_{\mathrm{gt}}\right)=\frac{1}{|P_{\mathrm{pred}}|}\sum_{p\in P_{\mathrm{pred}}}\min_{q\in P_{\mathrm{gt}}}{∥p-q∥}_{2}^{2}+\frac{1}{|P_{\mathrm{gt}}|}\sum_{q\in P_{\mathrm{gt}}}\min_{p\in P_{\mathrm{pred}}}{∥q-p∥}_{2}^{2}.$$

This captures both under-prediction and over-extension in the predicted graph structure.

#### 4.3.2. Node and Edge Count

To evaluate topological correctness, we compare the number of key nodes and edges between the predicted and ground-truth graphs. We simplify each graph by removing intermediate points on straight-line segments and retaining only nodes with degree≠2 (i.e., endpoints and branching points). The resulting simplified graph is denoted $G_{\mathrm{simp}}$, and we report the total number of nodes and edges in this graph as structural indicators. A well-predicted graph should match the ground truth in both key node count and structural connectivity.

#### 4.3.3. Tree Rate

To evaluate how well the output graph satisfies the constraint, we calculate the probability that the output graph forms a tree structure. We consider a graph as a tree if it is connected and contains no cycles, i.e., |E|=|V|−1.

### 4.4. Baselines

To ensure a comprehensive evaluation, we compare our method with two representative baselines: geometry-based and learning-based methods.

#### 4.4.1. adTree

**Figure 5:**
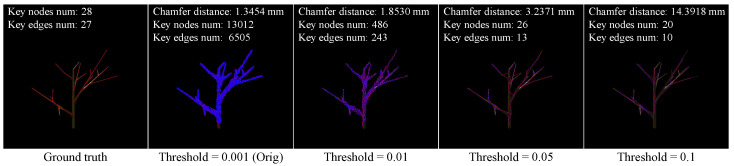
Effect of duplication removal threshold in AdTree. Visual comparison under varying thresholds illustrates that the default value (0.001) leads to excessive node retention and an over-connected graph, artificially lowering the Chamfer score. A high threshold (0.1) oversimplifies the structure, while a moderate threshold (0.05) yields a result close to the ground truth with better structural fidelity (Red lines and blue dots denote the edges and nodes of the tree graph, respectively).

adTree [24] is a classical geometry-based pipeline for accurate and automatic 3D tree reconstruction. AdTree extracts an initial skeleton by building an MST based on Dijkstra’s shortest paths. It iteratively prunes redundant or noisy components, and cylinders are fitted through an optimization process to approximate branch geometry. As a purely geometric method, adTree does not rely on any learned parameters, making it robust to unseen species but sensitive to the completeness and density of the input point cloud. In practice, the default duplicate removal threshold (0.001) retains too many noisy points, leading to severely over-connected graphs (13,012 nodes vs. 28 in the ground truth). A higher threshold, like 0.1, oversimplifies the structure. Following careful tuning, we adopt 0.05, which generates a reasonable 26-node skeleton close to the ground truth, as shown in Figure 5.

#### 4.4.2. Smart-Tree

Smart-Tree [26] represents a recent learning-based method that approximates branch skeletons from point clouds using a supervised approach. It employs a sparse voxel convolutional neural network to predict each point’s radius and medial axis direction, followed by a greedy post-processing algorithm to extract the final skeleton. Unlike geometry-based methods that require species-specific parameter tuning, Smart-Tree learns structural priors from data and generalizes across diverse tree morphologies. However, it relies on large-scale supervised training and may degrade under domain shift.

To ensure a fair comparison, we normalize the input conditions across all methods. Specifically, we transform our predicted point cloud into the same upright orientation as the baselines and rescale it to match the overall bounding box dimensions of each baseline’s output. This process reduces discrepancies on a global scale and orientation, ensuring that structural evaluation focuses on skeleton quality rather than external input variation.

### 4.5. Results on the Synthetic Dataset

We evaluate all methods on two synthetic datasets with distinct structural properties: the L-system-based and Mtree datasets. Table 1 presents quantitative comparisons.

#### 4.5.1. L-System-Based Dataset

**Figure 6:**
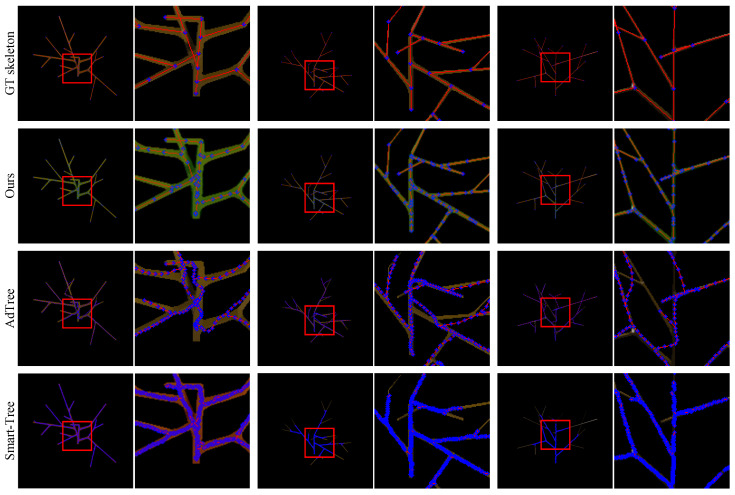
Qualitative results on the L-system-based dataset. From top to bottom: ground-truth skeletons, our method, AdTree, and Smart-Tree. We overlay green MST-based mask contours to visualize the alignment between predicted radii and ground-truth structure. Our method consistently achieves the most accurate and well-aligned predictions. AdTree tends to overfit straight structures with curved paths, while Smart-Tree suffers from over-segmentation and fails to reconstruct thin branches, despite its high point density (Red lines and blue dots denote the edges and nodes of the tree graph, respectively).

Figure 6 presents qualitative comparisons on the L-system-based dataset. From top to bottom, we show the ground-truth skeletons and results from our method, AdTree, and Smart-Tree. Visually, our method exhibits the closest alignment with the green masks in branch radii and node placement.

AdTree tends to generate overly curved structures, even when the underlying tree shape is relatively straight, which introduces geometric bias. Although Smart-Tree can produce dense point distributions, it often fails to capture thin branches, leading to incomplete skeletons and high Chamfer distance variance. Quantitatively, our method achieves the lowest average and standard deviation in Chamfer distance (0.19±0.20 mm) and a minor error in node count. AdTree also performs relatively well in terms of connectivity, while SmartTree suffers from inconsistent predictions. Furthermore, ours is the only method that yields a valid tree in every test case (Tree Rate 100%), while both baselines produce graphs with cycles or disconnected parts (Tree Rate 0%).

#### 4.5.2. Mtree Dataset

**Figure 7:**
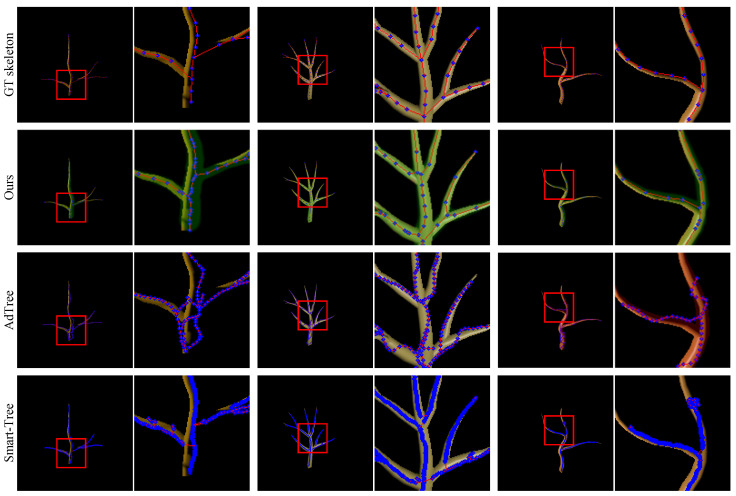
Qualitative results on the Mtree dataset. From top to bottom: ground-truth skeletons, our method, AdTree, and Smart-Tree. As with the L-system results, green MST-based masks highlight branch regions. While all methods show similar trends, the increased curvature and diameter variation in Mtree trees amplify the weaknesses of AdTree and Smart-Tree. Our method remains the most faithful to the ground truth in both geometry and structure (Red lines and blue dots denote the edges and nodes of the tree graph, respectively).

Figure 7 shows the more challenging Mtree dataset results. AdTree continues producing geometrically biased, over-curved structures due to its reliance on global point trends that can smooth nearby branches. Smart-Tree performs better overall but often fails to recover thin terminal branches.

Our method remains robust across these conditions, producing structurally complete skeletons and accurately modeling branch radii variation. We again achieve the lowest Chamfer distance (15.66±8.12 cm) and perfect tree rate (100%), validating the strength of our mask-guided refinement and structure-aware graph optimization.

### 4.6. Results on the Real-World Dataset

**Figure 8:**
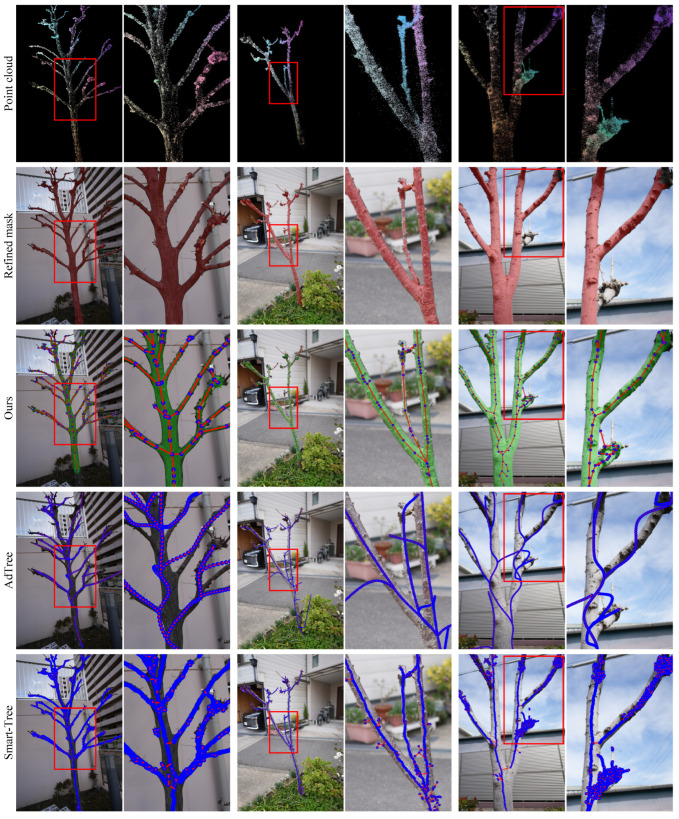
Qualitative results on the real-world dataset. From top to bottom: point cloud, refined mask, our method, AdTree, and Smart-Tree. We overlay green MST-based mask contours to visualize the alignment between predicted radii and ground-truth structure. To enhance clarity, the visualization thickness of nodes (blue) and edges (red) is adjusted based on tree species.

**Figure 9:**
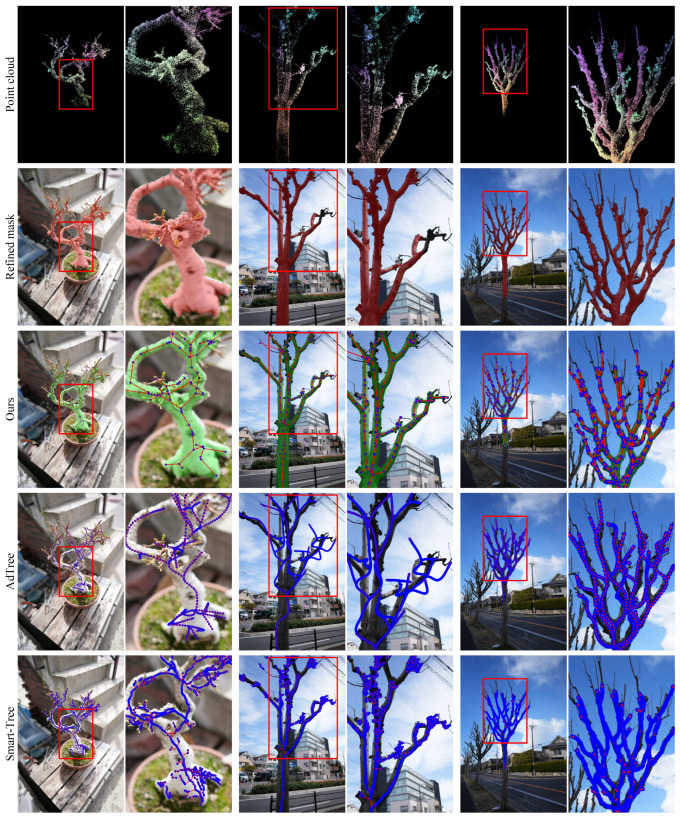
Qualitative results on the real-world dataset. From top to bottom: point cloud, refined mask, our method, AdTree, and Smart-Tree. We overlay green MST-based mask contours to visualize the alignment between predicted radii and ground-truth structure. To enhance clarity, the visualization thickness of nodes (blue) and edges (red) is adjusted based on tree species.

We evaluate all methods on six real-world trees, including five common urban landscaping species and one potted plant. As shown in Figure 8 and Figure 9, the visual complexity of natural scenes, such as occlusion, irregular branching, and thin structures, amplifies the limitations of baseline methods.

AdTree continues to produce overly curved branches, especially in trunk-adjacent regions, as it globally fits trends in the point cloud and often merges nearby but distinct branches into a single curved trajectory. Smart-Tree, which is based on clustering, frequently produces disconnected components rather than coherent tree-like structures. Moreover, continuous geometry is often over-segmented due to local variations in point density along a single branch. It introduces spurious thin sub-branches, especially in denser regions, resulting in structural noise even along otherwise straight branches.

In addition, we observe that certain anatomical features, such as nodal swellings, galls, or terminal ends of branches, often receive disproportionately dense matches during MVS reconstruction. These localized point cloud accumulations are not representative of true geometric importance. However, they pose significant challenges for baseline methods: AdTree tends to overfit these regions, generating undesired curves near trunk junctions, while Smart-Tree often segments them into densely stacked components, introducing clutter and misinterpreted sub-structures.

In contrast, our method maintains structural connectivity and faithfully reconstructs branching geometry. Notably, even when refined 2D masks (shown in red) are incomplete in certain views, our model concludes plausible geometry by integrating the multi-view masks’ distribution through the soft blur of 3DGS. All reconstructed graphs are valid trees (tree rate 100%), and the alignment between predicted radii and the overlaid green MST masks confirms high fidelity in topology and shape.

### 4.7. Ablation Study

**Figure 10:**
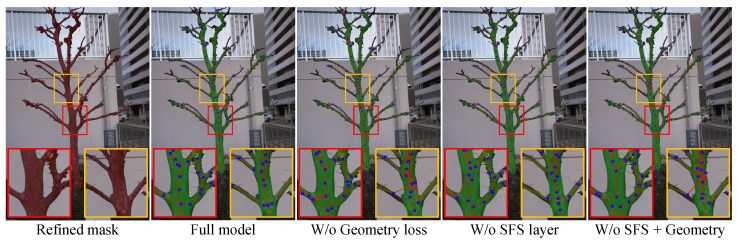
Ablation study on loss components. From left to right: refined mask, full model (ours), without $L_{\mathrm{graph}}$, without the SFS layer, and without both. All results are visualized on the same real-world scene with the predicted skeleton overlaid in green. Removing geometry regularization causes jagged edges and sharp angles, while removing the SFS layer disrupts edge connectivity realism. Removing both leads to severe geometry and topology degradation, confirming the importance of each loss term (Red lines and blue dots denote the edges and nodes of the tree graph, respectively).

We conduct controlled ablations to isolate the effect of each major loss term. As shown in Figure 10, removing any of the two key objectives leads to degraded geometry and topology.

#### 4.7.1. Without Geometry Regularization ($L_{\mathrm{graph}}$)

Disabling the graph geometry regularization leads to unnatural tree-like geometry. In particular, without constraints on edge length, angle smoothness, or directional spacing, the reconstructed graph loses key features in real plants, resulting in unnatural zig-zagging edges, sharp angular bends, and uneven node spacing.

#### 4.7.2. Without an SFS Layer

The SFS layer softly reweights the predicted adjacency matrix to align with the MST structure. Unlike the geometric artifacts caused by removing $L_{\mathrm{graph}}$, which disrupts local angles and spacing, removing the SFS layer leads to incorrect edge connectivity. This topological inconsistency undermines the plausibility of branch bifurcations and the overall tree structure.

#### 4.7.3. Without an SFS Layer and Geometry Regularization

Removing both the SFS layer and $L_{\mathrm{graph}}$ combines the weaknesses of topological and geometric inconsistency. The predicted graph exhibits misplaced connections, zig-zagging edges, sharp angles, and uneven spacing. Without any structural prior, the graph fails to capture both the physical realism and the edge-connect logic of natural trees.

## 5. Conclusions

We introduced a differentiable graph optimization framework for reconstructing 3D tree skeletons from multi-view segmentation masks. Our method jointly refines node locations, branch radii, and edge probabilities by coupling an MST-initialized graph with 3D Gaussian rendering. Thanks to the parameterization of the adjacency matrix and the mask-guided optimization, every prediction yields a valid tree structure. On both synthetic and real-world datasets, we achieve a 100% tree rate while significantly reducing Chamfer distance, node count error, and edge error compared to AdTree and Smart-Tree. The soft blur in 3DGS makes our approach robust to pose noise and partial mask inaccuracies.

### Limitations and Future Work

Our datasets contain bare or lightly occluded branches. Heavy foliage may degrade mask quality and, by extension, silhouette supervision. We plan to collect leaf-on scenes and investigate joint leaf/branch segmentation to maintain accuracy under severe occlusion. Compared with purely geometric pipelines like AdTree, our mask-guided refinement adds roughly five minutes of optimization per tree on a single RTX 4090. Although acceptable for offline processing, this overhead prevents real-time deployment. Future work will explore incremental optimization and a lightweight neural renderer to shorten convergence time.

## Figures and Tables

**Table 1:** Quantitative evaluation on synthetic datasets (Bold numbers indicate the best scores). The Chamfer distance measures geometric similarity. Δ|E| and Δ|N| represent the absolute differences in the number of predicted edges and nodes relative to the ground-truth counts (average graph sizes: 29.32 key nodes and 28.32 key edges for the L-system-based dataset; 15.36 key nodes and 14.40 key edges for the Mtree dataset).

Method	L-System-Based Dataset				Mtree Dataset			
	Chamfer Distance	Δ|E|↓	Δ|N|↓	Tree Rate	Chamfer Distance	Δ|E|↓	Δ|N|↓	Tree Rate
	(mm) ↓			%↓	(cm) ↓			%↓
AdTree	3.06 ± 0.68	14.18	1.04	0.0	53.91 ± 79.19	**1.52**	10.40	0.0
Smart-Tree	8.86 ± 16.66	2.22	22.28	0.0	31.15 ± 76.69	3.66	20.76	0.0
Ours	**0.19 ± 0.20**	**0.22**	**0.22**	**100.0**	**15.66 ± 8.12**	2.28	**2.24**	**100.0**

## Data Availability

The datasets presented in this study are not readily available because they are currently being used in the preparation of other manuscripts. Requests to access the datasets should be directed to the corresponding author.
